# Preliminary evaluation of dual-energy CT to quantitatively assess bone marrow edema in patients with diabetic foot ulcers and suspected osteomyelitis

**DOI:** 10.1007/s00330-023-09479-2

**Published:** 2023-02-23

**Authors:** M. A. Mens, A. de Geus, R. H. H. Wellenberg, G. J. Streekstra, N. L. Weil, S. A. Bus, T. E. Busch-Westbroek, M. Nieuwdorp, M. Maas

**Affiliations:** 1 Radiology and Nuclear Medicine, Amsterdam UMC, Location University of Amsterdam, Meibergdreef 9, 1105 AZ Amsterdam, The Netherlands; 2 Amsterdam Movement Sciences, Rehabilitation and Development, Amsterdam, The Netherlands; 3 Biomedical Engineering and Physics, Amsterdam UMC, Location University of Amsterdam, Meibergdreef 9, Amsterdam, The Netherlands; 4 Rehabilitation Medicine, Amsterdam UMC, Location University of Amsterdam, Meibergdreef 9, Amsterdam, The Netherlands; 5 Internal Medicine, Amsterdam UMC, Location University of Amsterdam, Meibergdreef 9, Amsterdam, The Netherlands; 6 Amsterdam Cardiovascular Sciences, Diabetes and Metabolism, Amsterdam, The Netherlands

**Keywords:** Bone marrow, Edema, Osteomyelitis, Diabetic foot, X-ray computed tomography

## Abstract

**Objectives:**

The purpose of this study is to evaluate the value of dual-energy CT (DECT) with virtual non-calcium (VNCa) in quantitatively assessing the presence of bone marrow edema (BME) in patients with diabetic foot ulcers and suspected osteomyelitis.

**Methods:**

Patients with a diabetic foot ulcer and suspected osteomyelitis that underwent DECT (80 kVp/Sn150 kVp) with VNCa were retrospectively included. Two observers independently measured CT values of the bone adjacent to the ulcer and a reference bone not related to the ulcer. The patients were divided into two clinical groups, osteomyelitis or no-osteomyelitis, based on the final diagnosis by the treating physicians.

**Results:**

A total of 56 foot ulcers were identified of which 23 were included in the osteomyelitis group. The mean CT value at the ulcer location was significantly higher in the osteomyelitis group (− 17.23 ± 34.96 HU) compared to the no-osteomyelitis group (− 69.34 ± 49.40 HU; *p* < 0.001). Within the osteomyelitis group, the difference between affected bone and reference bone was statistically significant (*p* < 0.001), which was not the case in the group without osteomyelitis (*p* = 0.052). The observer agreement was good for affected bone measurements (ICC = 0.858) and moderate for reference bone measurements (ICC = 0.675). With a cut-off value of − 40.1 HU, sensitivity was 87.0%, specificity was 72.7%, PPV was 69.0%, and NPV was 88.9%.

**Conclusion:**

DECT with VNCa has a potential value for quantitatively assessing the presence of BME in patients with diabetic foot ulcers and suspected osteomyelitis.

**Key Points:**

• *Dual-energy CT (DECT) with virtual non-calcium (VNCa) is promising for detecting bone marrow edema in the case of diabetic foot ulcers with suspected osteomyelitis*.

• *DECT with VNCa has the potential to become a more practical alternative to MRI in assessing the presence of bone marrow edema in suspected osteomyelitis when radiographs are not sufficient to form a diagnosis*.

## Introduction

Diabetic foot ulcers are common complications of diabetic peripheral polyneuropathy. The prevalence of diabetic foot ulcers is 6.3% and treatment of these ulcers requires a multidisciplinary approach [1, 2]. They can become infected and lead to osteomyelitis. Osteomyelitis is difficult to treat and may require hospitalization for intravenous antibiotics [3]. If the infection does not respond to antibiotics and debridement, amputation might be needed. Amputations decrease quality of life but are often the only remaining option [4]. An early diagnosis is necessary to prevent this is needed. However, diagnosing osteomyelitis is complex [5, 6]. A combination of clinical examination and diagnostics such as wound or bone cultures and imaging is used. The International Working Group on the Diabetic Foot recommends a plain radiograph as the initial form of imaging [3]. Plain radiographs are widely available and inexpensive. If characteristic signs of osteomyelitis are present, the diagnosis is very likely. However, in the first few weeks, a plain radiograph is often negative [3]. If a plain radiograph does not suffice, the next option is Magnetic Resonance Imaging (MRI). MRI enables reliable detection of bone marrow edema (BME), an indicator that osteomyelitis might be present [7]. However, MRI is expensive, has a long acquisition time, and cannot be used in patients with certain implants, pacemakers, or claustrophobia.

Dual-energy computed tomography (DECT) has been shown to be an alternative to MRI in BME detection [8–19]. DECT is less expensive, has a shorter acquisition time, and has no contraindications. This form of CT enables tissue differentiation based on attenuation properties at different tube voltages [20, 21]. Using a virtual non-calcium (VNCa) post-processing algorithm, differentiation between BME and healthy bone marrow is possible. Healthy bone marrow contains mostly fat which has lower CT values in Hounsfield units (HU) than water, which is present in BME.

Studies proposing cutoff values for BME range between − 80 HU and 35 HU [8–19]. These studies were conducted to detect BME in traumatic injuries such as fractures in the wrist, ankle, and vertebrae [12, 13, 15, 16, 18, 22]. DECT has also been investigated in forms of arthritis [23, 24]. However, there are no studies examining DECT for BME detection in osteomyelitis or diabetic foot disease. If DECT proves to be able to detect BME in this patient group (i.e., diagnose osteomyelitis in an early stage, diagnose osteomyelitis in difficult cases, and evaluate treatments of osteomyelitis), DECT would be a valuable addition to the radiological process. Therefore, this study aims to assess the applicability and reproducibility of DECT with VNCa for detecting BME in patients with diabetic foot ulcers and suspected osteomyelitis.

## Materials and methods

### Study population

All patients being treated at the diabetic foot expert center of our tertiary referral hospital who had undergone a DECT scan of the feet between January 2018 and January 2021 were retrospectively selected and screened. Inclusion criteria were clinically suspected osteomyelitis and an open ulcer at the time of scanning. Patients were excluded in the case of concomitant foot diseases (e.g., active Charcot osteoarthropathy or recent trauma) on the foot with the ulcer of interest and if the patients’ digital files were unavailable. Patients were divided into two clinical study groups: osteomyelitis and no-osteomyelitis depending on the final clinical diagnosis. This diagnosis was based on clinical data and findings during follow-up including depth of the ulcer (i.e., bone contact), fever, laboratory values indicating inflammation, MRI findings, bone biopsy findings, ulcer progression or healing, amputation, and structural changes on the weighted average or mixed images of the CT scans (CT was not used to evaluate BME for clinical diagnosis). The Medical Ethics Review Committee provided a waiver for this study.

### DECT protocol

All DECT scans were performed using a dual-source CT scanner (SOMATOM Force; Siemens Healthcare) using 80 kVp (tube A) and 150 kVp with Sn filtration (tube B). Automatic attenuation-based tube current modulation was applied on both tubes with a reference mAs of 150 for tube A and a reference mAs of 380 for tube B. Other CT parameters were a Qr54d or Qr40d kernel (medium smooth), rotation time of 0.5 s, collimation of 0.6 mm, slice thickness of 1.5 mm, increment of 1.5 mm, mean CT dose index volume (CTDIvol) of 6.0 mGy (range 4.1–6.6 mGy), and mean dose length product (DLP) of 152.8 mGy/cm (range 97.0–206.4 mGy/cm).

### DECT post-processing

After each DECT acquisition, three datasets were reconstructed: an 80-kVp dataset, an Sn150-kVp dataset, and a weighted average or mixed dataset from both tubes. The weighted average was used to simulate and replace a conventional CT scan and was used in clinical practice. The scans were analyzed using the BME application in SyngoVia post-processing software (SyngoVia VB40; Siemens Healthcare) with a threshold of 100 HU and a maximum of 800 HU. Using a three-material decomposition algorithm, yellow bone marrow, red bone marrow, and bone mineral could be differentiated, allowing for subtraction of calcium and the creation of VNCa images. The default settings for color-coded maps of bone marrow with densities between − 150 HU and 100 HU were used.

### Image analysis

**Fig. 1:**
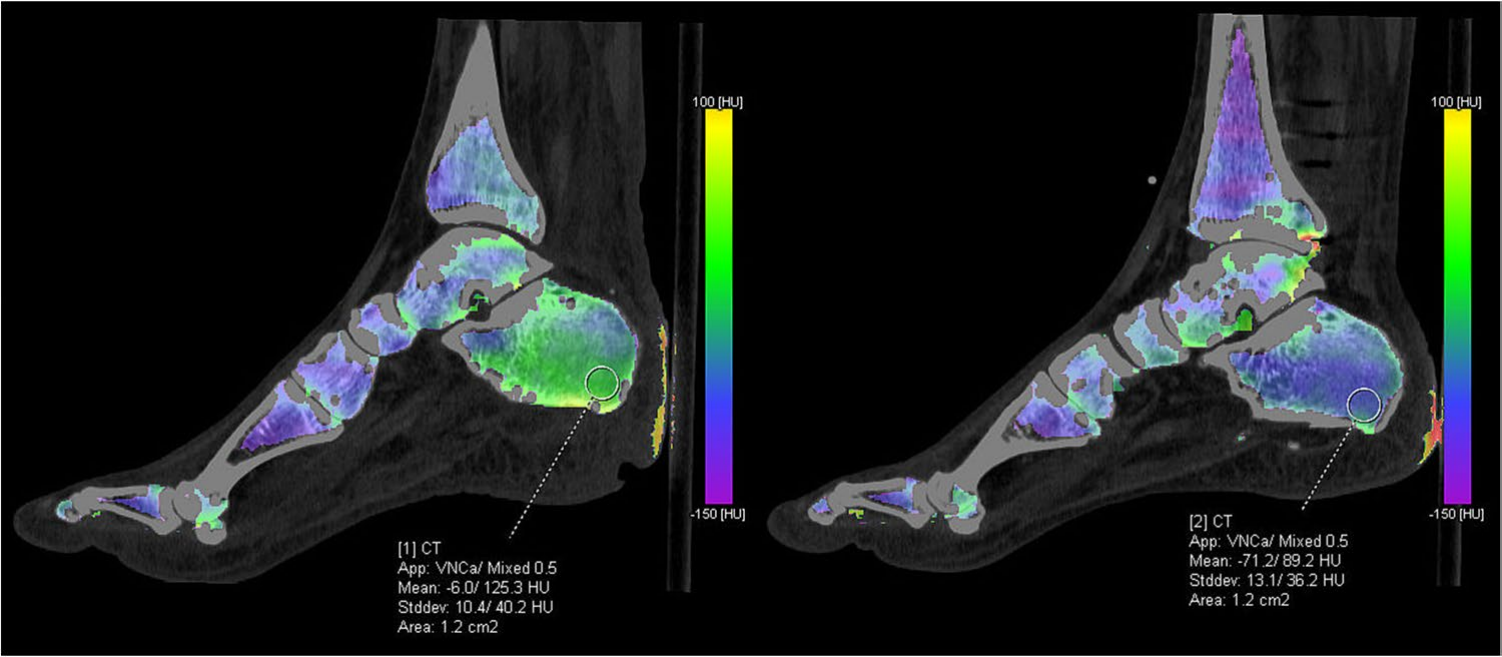
Measurement of CT values in the calcaneus on the ulcer side (left) and the reference side (right)

**Fig. 2:**
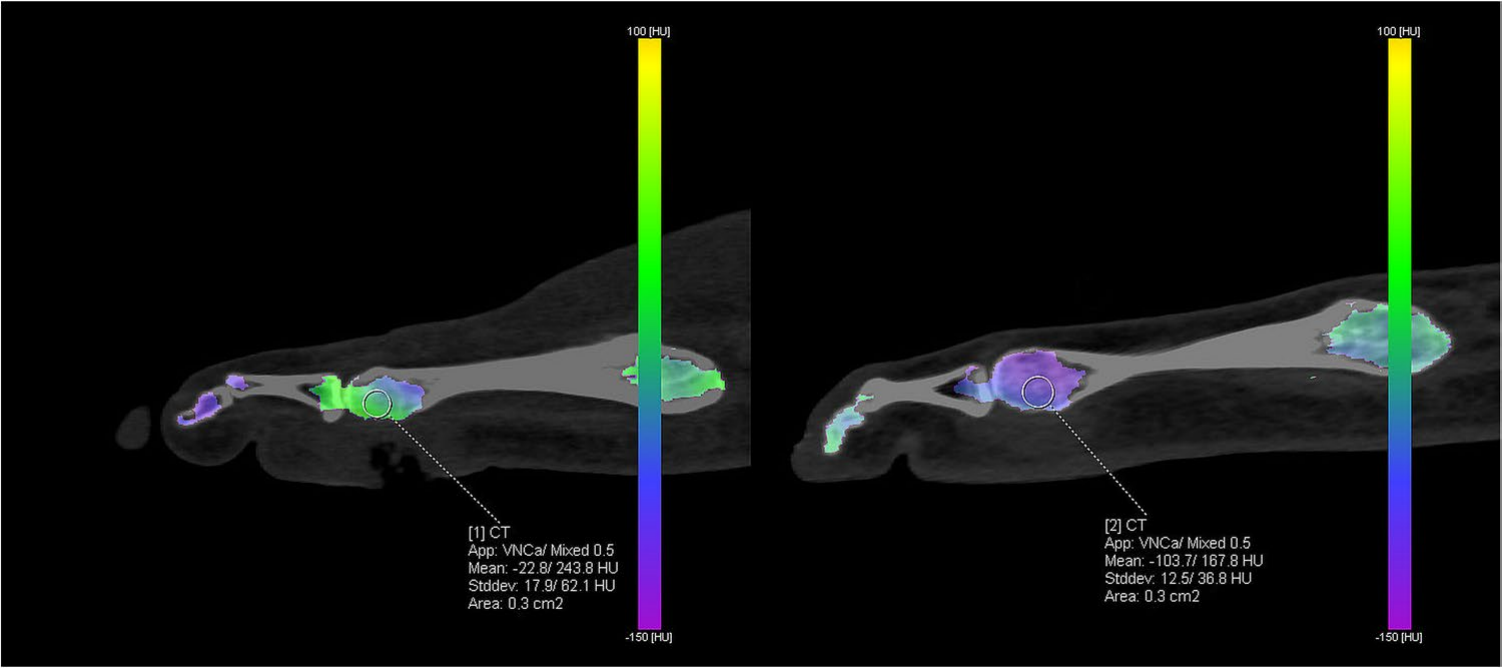
Measurement of CT values in the fifth metatarsal head on the ulcer side (left) and the reference side (right)

All scans were independently assessed by two investigators (M.A.M. and A.G.) who were blinded for the radiology report. The ulcer was identified on the weighted average DECT dataset using clinical information related to ulcer location. In difficult cases, an experienced musculoskeletal radiologist (M.M.) assisted in locating the ulcer. A circular region of interest (ROI) was manually placed in sagittal or coronal VNCa images within bones with suspected osteomyelitis as close as possible to the index ulcer (Figs. 1 and 2). If multiple ulcers with suspected osteomyelitis were present, all ulcers were assessed. If the bone of interest was too small to place a circular ROI, a freehand ROI was drawn as large as possible without including irrelevant structures. On the same location in the contralateral foot, a reference ROI with the same surface area as the ROI in the bone of interest was drawn. If this location was not suitable as a reference (e.g., due to amputation, Charcot, ulceration, or logistics), the talus of the contralateral or (if the contralateral talus was also not suitable) the ipsilateral talus was used as a reference. The CT values of the ROI in the affected bone and the reference bone were measured. If CT values differed more than 50 HU between observers, an experienced musculoskeletal radiologist (M.M.) performed a third measurement. This measurement was used in further calculations, except for calculations related to observer variability. Measurements with less than 50 HU difference between observers were reviewed by two radiologists (M.M. and N.L.W.) with, respectively, 30 years and 5 years of experience in musculoskeletal radiology. Additionally, they independently and qualitatively assessed the cases for presence of BME. In cases where minimally one radiologist considered BME to be present, a positive finding of BME was used for further analysis. BME has CT values of approximately 0 and will be displayed as green on the color-coded map. Higher CT values are more yellow or eventually red and lower CT values are blue or purple (Figs. 1 and 2).

### Statistical analysis

Descriptive statistics were used to describe differences in demographics between groups. Data regarding CT values were tested for normality using the Kolmogorov–Smirnov test. An independent-samples *t*-test was used to test for significance between the osteomyelitis and no-osteomyelitis group regarding CT values on both the affected and reference side and a chi-squared test was used to test for significance in the qualitative analysis. The difference between the affected side and reference side was tested for significance using a paired-samples *t*-test. A *p* < 0.05 was considered statistically significant. Inter-observer agreement was determined by calculating the intraclass correlation coefficient (ICC) or Cohen’s kappa. An ICC < 0.5 was considered “poor”; 0.5–0.74, “moderate”’ 0.75–0, “good”; and > 0.9, “excellent” [25]. A kappa < 0 was considered “poor”; 0–0.20, “slight”; 0.21–0.40, “fair”; 0.41–0.60, “moderate”; 0.61–0.80, “substantial”; and > 0.81, “almost perfect” [26]. Additionally, Bland–Altman plots with limits of agreement were obtained. Using receiver operating characteristic (ROC) analysis and Youden’s index, a cutoff CT value for determining BME was proposed. Sensitivity, specificity, positive predictive value (PPV), and negative predictive value (NPV) were calculated. All statistical tests were performed using SPSS (version 26.0, released 2019, IBM SPSS Statistics for Windows; IBM Corporation).

## Results

DECT scans of 56 ulcers in 37 patients were identified and analyzed. These patients consisted of 29 men and 8 women with a mean age of 61.7 ± 12.6 years. There were no significant differences in demographics between the clinical osteomyelitis and clinical no-osteomyelitis group (Table 1). Of the 56 ulcers, 20 (36%) were located on the plantar side of the foot. Twenty-two (39%) of all ulcers were located on either the hallux or underneath the first metatarsal head, followed by 9 (16%) ulcers located underneath the fifth metatarsal head and 7 (13%) located on the calcaneus. A wound swab culture was performed in 49 of 56 ulcers (88%). *Staphylococcus aureus* was the most prevalent pathogen (*N* = 22; 45%) followed by group G *Streptococcus* (*N* = 5; 10%). The wound culture did not show any growth in 11 ulcers (22%).

**Table 1:** Patient demographics

Ulcers (*N*)	56
Patients (*N*)	37
Age (years)	61.7 ± 12.6
Sex (men:women)	29:8
Height (cm)	177.5 ± 9.4
Weight (kg)	94.1 ± 17.1
Body mass index (kg/m^2^)	30.1 ± 5.1

### Ulcer outcomes

Osteomyelitis was clinically diagnosed in 23 of 56 ulcers. There was an amputation in 9 cases and 2 patients died before the ulcer was closed in the osteomyelitis group. Of the remaining 12 ulcers in this group, the mean time until closing was 18.5 ± 14.7 weeks. There was no clinical indication of osteomyelitis in 33 of the 56 ulcers. Amputation was performed in 10 cases before the ulcer was closed in the no-osteomyelitis group and there were no deaths before ulcers closed. The remaining 23 ulcers in this group had a mean time until closing of 16.3 ± 20.3 weeks.

### Quantitative analysis

**Fig. 3:**
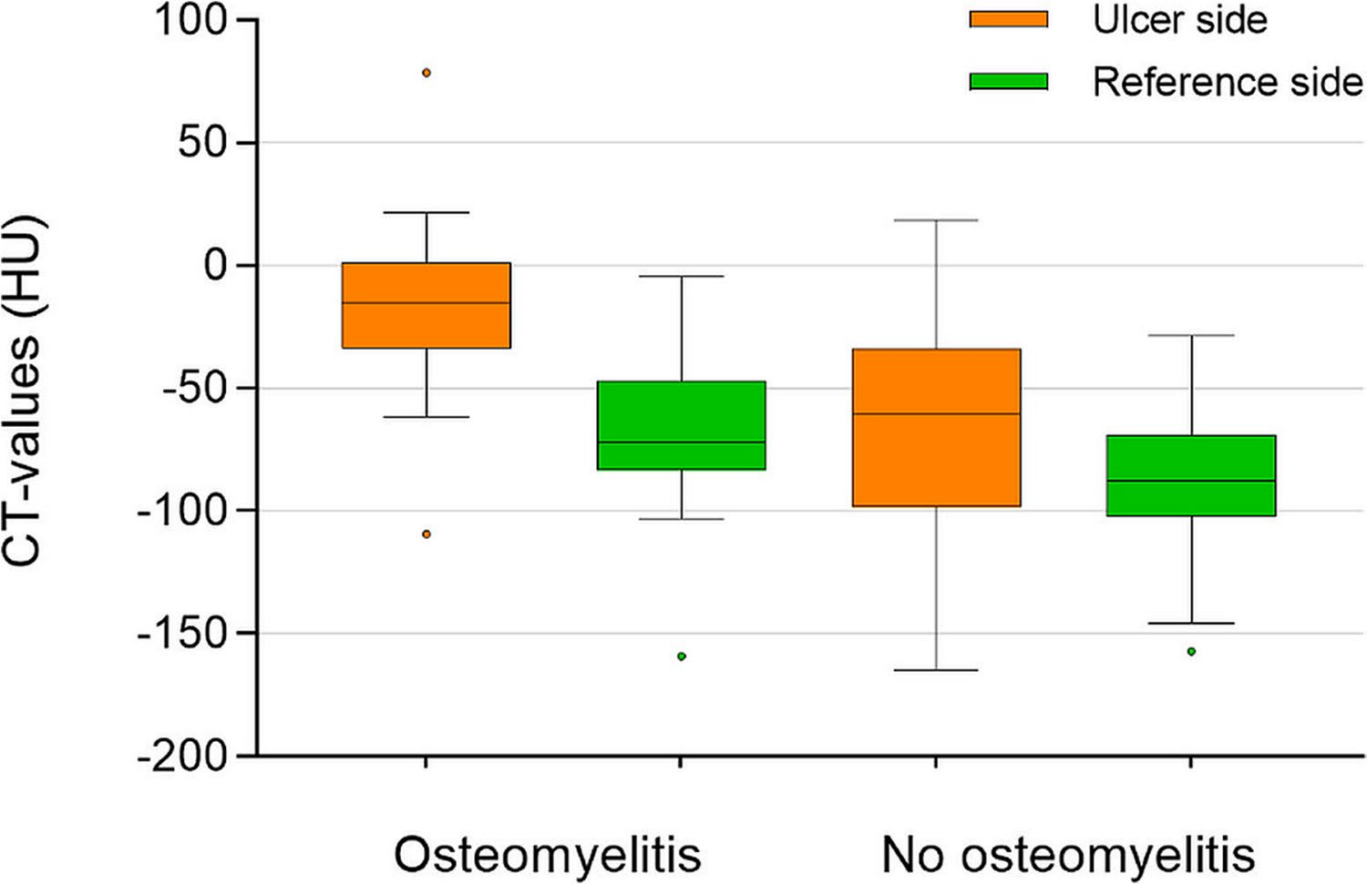
Box plots showing the CT values on the ulcer and reference location in the two groups (osteomyelitis and no-osteomyelitis)

**Fig. 4:**
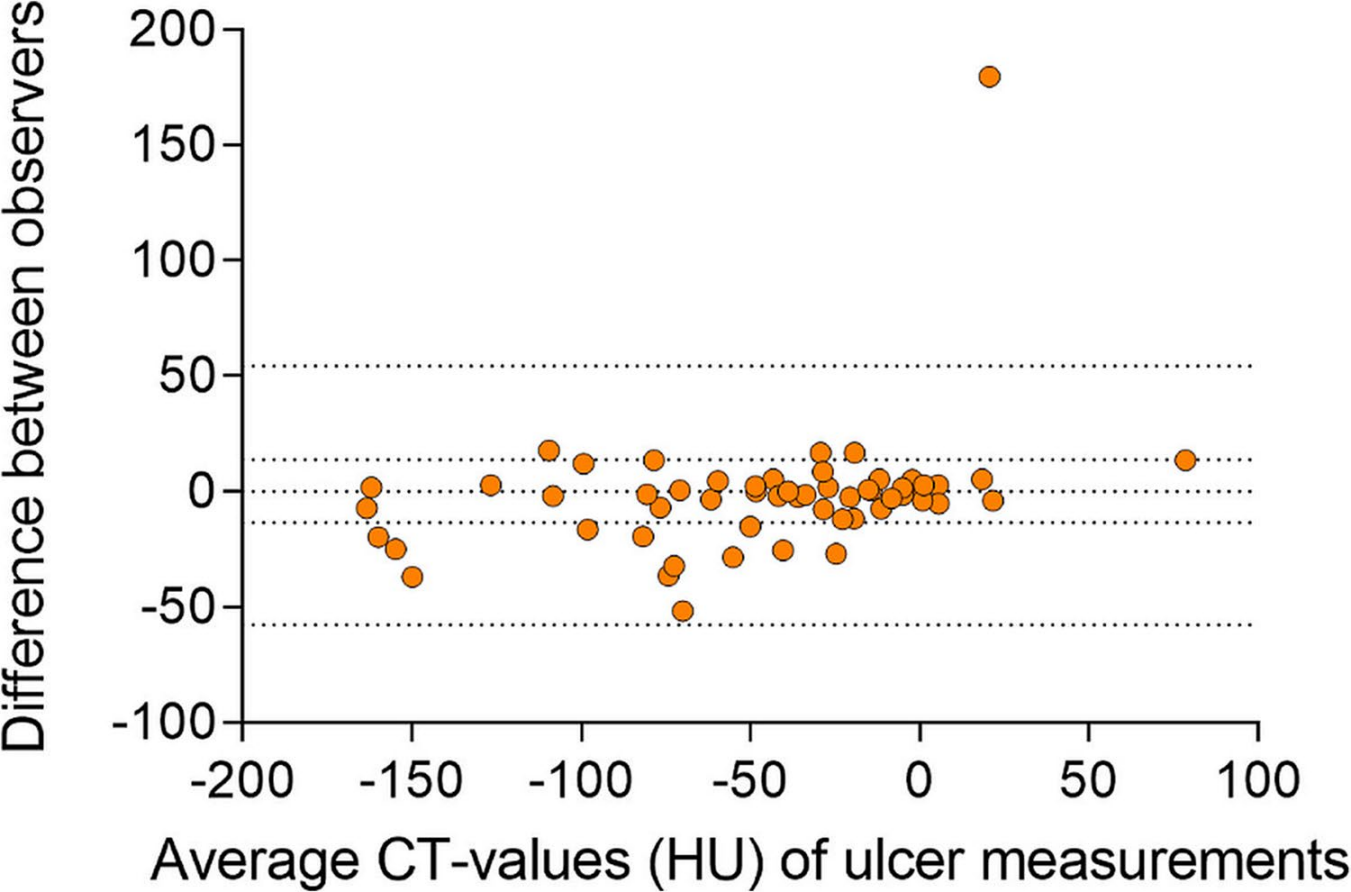
Bland–Altman plot of the difference between the two observers of the measurements on the ulcer side

**Fig. 5:**
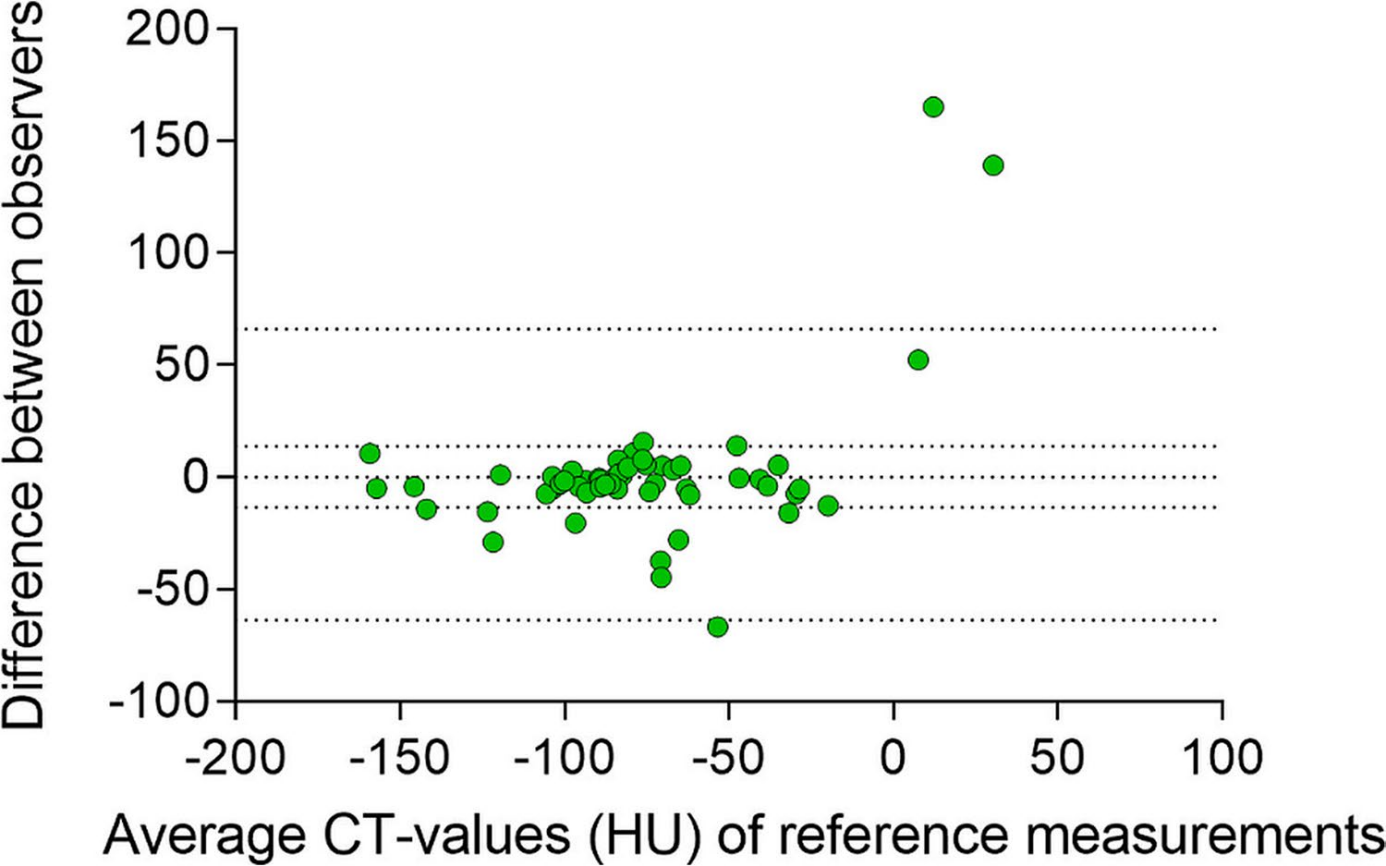
Bland–Altman plot of the difference between the two observers of the measurements on the reference side

**Fig. 6:**
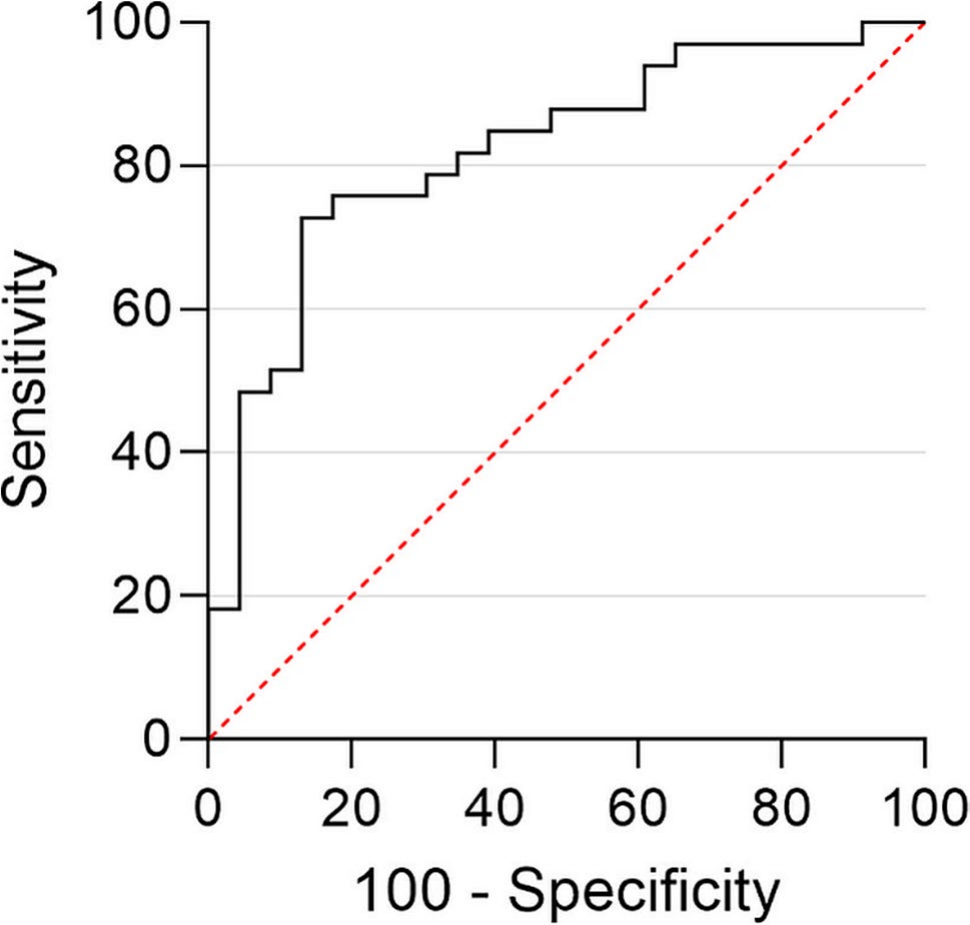
Receiver operating characteristic (ROC) curves of the CT values. The area under the curve was 0.821

There was a significant difference in CT values of the bone marrow in the bone adjacent to the ulcer between the osteomyelitis and no-osteomyelitis groups (− 17.23 ± 34.96 HU and − 69.34 ± 49.40 HU, *p* < 0.001) (Fig. 3). The CT values of the reference location did not differ significantly between the two groups (− 68.90 ± 33.09 HU and − 86.26 ± 32.00 HU, *p* = 0.054) (Fig. 3). Regarding the osteomyelitis group, there was a significant difference between the ulcer location and reference location (*p* < 0.001). In the no-osteomyelitis group, the ulcer location showed similar CT values to the reference location (*p* = 0.052). The inter-observer agreement was “good” for measurements on the ulcer location (ICC = 0.858) and “moderate” for the reference measurements (ICC = 0.675). Bland–Altman plots were made to represent the difference between observers on the ulcer side (Fig. 4) and reference side (Fig. 5). There were 6 measurements with a difference between the observers of more than 50 HU of which 2 on the ulcer side. These 6 measurements were repeated by the third observer (M.M.). The original measurements (by M.A.M. and A.G.) are presented in the Bland–Altman plots (Figs. 4 and 5). The radiologists agreed with 96% of the other measurements. In the remaining 4%, the radiologists would have placed the ROI further from the cortex and they would have excluded one case due to metal artifacts. However, changing the ROI placement would not have influenced the results. On the ulcer side, there was a bias of − 1.69 with an SD of 28.5. The 95% agreement limits were − 57.6 and 54.3. On the reference side, the bias was 1.30 with an SD of 33.4 and 95% agreement limits of − 64.2 and 66.8. The area under the ROC curve was 0.821 (Fig. 6). With a cutoff value of − 40.1 HU, sensitivity was 87.0%, specificity was 72.7%, PPV was 69.0%, and NPV was 88.9%.

### Qualitative analysis

BME was marked as present in 37 of the 56 cases (66.1%). There was a significant difference between the osteomyelitis and no-osteomyelitis groups in BME presence (*p* = 0.006). There was disagreement in 2 cases and Cohen’s kappa was “almost perfect” (*κ* = 0.89). Sensitivity was 87.0%, specificity was 48.5%, PPV was 54.1%, and NPV was 84.2%.

## Discussion

This retrospective study aimed to quantitatively assess the use of DECT with VNCa in diagnosing osteomyelitis due to diabetic foot ulcers by detecting BME. The group with osteomyelitis showed significantly higher CT values in the bone marrow close to the ulcer than the group without osteomyelitis. Furthermore, the CT values in the bone marrow with suspected osteomyelitis were significantly higher than the CT values in the bone marrow of the reference bone in the same participant. This indicates that DECT with VNCa is a promising form of imaging to detect BME in patients with diabetic foot ulcers and suspected osteomyelitis.

Using a cutoff value of − 40.1 HU, a sensitivity of 87.0% and a specificity of 72.7% were acquired using quantitative measurement. Regarding the qualitative analysis, a sensitivity of 87.0% and a specificity of 48.5% were found. The relatively low specificity can be explained by the fact that the color-coded overlay exaggerated small rises in clinically irrelevant CT values. In addition, rises in CT values due to stress reactions were flagged as pathological.

In studies focusing on BME detection using DECT with VNCa in other skeletal pathologies, the diagnostic performance was higher than in our study. In vertebral fractures, a pooled estimate of sensitivity and specificity of 89% and 95%, respectively, was reported [26]. In the appendicular skeleton, a sensitivity of 86% and specificity of 93% were found. Osteomyelitis leads to both water influx and granulation tissue; therefore, higher diagnostic performance could be expected. One of the main differences between the studies included in these reviews and our study is that we studied BME due to infection. Most previous studies concerned traumatic injury. There are a few studies that quantitatively analyzed DECT to detect BME in non-traumatic diseases such as sacroiliitis [23, 24]. However, there have been no studies concerning infections.

Use of DECT with VNCa has been assessed in the calcaneus and talus but never in the midfoot and forefoot [14, 27]. The bones in the midfoot and forefoot are relatively small making it more difficult to acquire a good quantitative measurement. Additionally, it is possible that not all bones in the foot have the same CT values in a healthy condition. For instance, Guggerberger et al [14] showed different cutoff values for different parts of the talus. Therefore, it is not certain the same cutoff value can be used for every bone.

When assessing the increase in CT values to aid in the diagnosis of osteomyelitis, it has to be noted that this increase is not only due to water influx. In the process of osteomyelitis, there is loss of fatty bone marrow and influx of inflammatory tissue [27]. Fat depletion, visible as loss of T1-weighted signal, is used in MRI to diagnose osteomyelitis [7]. In DECT, fat depletion of bone marrow is an important factor that increases CT values.

We observed a relatively large variability in CT values in the group with an ulcer but without osteomyelitis and an overlap in CT values on the ulcer sides between the groups (Fig. 3). This can have multiple explanations. All patients in this study had diabetic peripheral polyneuropathy. This population can have BME without an acute cause [28, 29]. Additionally, other pathologies can cause BME such as Charcot osteoarthropathy or stress fractures. In our study, patients with a possibility to have these pathologies were excluded, but in clinical practice this needs to be kept in mind. However, osteomyelitis will remain the most likely diagnosis, if there is an ulcer and signs of infection.

There were some outliers in the Bland–Altman plots. These differences were due to mistakes in measurements and registration in data extraction tables. Two radiologists agreed with 96% of the other measurements and the Bland–Altman analysis showed a small bias of − 1.69. This shows that simple radiological measurements do not have to be performed by radiologists but can also be performed by trained personnel with lower qualifications. This fits in the trend of task shifting to reduce rising healthcare costs, waiting lists, and the reduction of workload of radiologists [30–32].

In the outpatient clinic, the diagnosis of osteomyelitis is primarily based on a combination of factors including the appearance of the ulcer, probe to bone test, (bone)culture, infection parameters, and imaging. Radiographs are the initial form of imaging performed [3]. A meta-analysis showed a sensitivity of around 69% and a specificity of 78% [33]. Thus, our quantitative results of DECT with VNCa generate a higher sensitivity than radiographs. These values are not as high as the sensitivity and specificity of MRI [3]. Therefore, with the present study, it is not possible to say if DECT could replace MRI. The downsides of using MRI in clinical practice are substantial. The long waiting time, contraindications for MRI, the discomfort for patients, and the costs already persuade physicians in our hospital to choose DECT with VNCa as an alternative to MRI. Therefore, our results indicating that DECT with VNCa is promising in this population are of importance for clinical practice.

A strength of the study is the use of quantitative measurements to assess areas with and without BME. The main limitation is the use of clinical diagnosis as a reference standard. Given the retrospective nature of this study, there was no MRI or bone biopsy available in most cases; thus, the clinical diagnosis and internal measurements in areas without BME were used as a reference standard. One could argue that the clinical diagnosis is a more accurate comparison than comparing with MRI, since all available clinical information is taken into account. Additionally, the exclusion of patients with concomitant foot disease could be a limitation.

Thus, DECT has a higher diagnostic accuracy in osteomyelitis imaging than conventional CT and radiography and is quicker, cheaper, and less burdensome for the patient than MRI. It is a possible alternative for MRI at negligible radiation dose. However, the accuracy of DECT in this patient group has to be further investigated, preferably in a prospective setting with MRI as a comparator.

In conclusion, DECT with VNCa is potentially valuable to quantitatively assess bone marrow changes in patients with diabetic foot ulcers and suspected osteomyelitis.

## Abbreviations

BME: Bone marrow edema
DECT: Dual-energy computed tomography
HU: Hounsfield units
ICC: Intraclass correlation coefficient
NPV: Negative predictive value
PPV: Positive predictive value
ROC: Receiver operating characteristic
ROI: Region of interest
VNCa: Virtual non-calcium

## Declarations

### Guarantor

The scientific guarantor of this publication is Prof. Dr. Mario Maas (Department of Radiology and Nuclear Medicine, Amsterdam University Medical Center, Amsterdam Movement Sciences).

### Statistics and biometry

No complex statistical methods were necessary for this paper.

### Methodology

• retrospective

• diagnostic or prognostic study

• performed at one institution
